# Tracheal reconstructive surgery under ECMO for the treatment of congenital tracheal stenosis in the premature infant: case report

**DOI:** 10.3389/fped.2024.1447418

**Published:** 2024-12-09

**Authors:** Qian Ya Xu, Tamang Sapana, Yu Qi, Guo Wei Fu, Long Hui Guo, Wei Ma, Li Li Wang, Gai Zhao, Hai Xia Wang, Qian Zhang

**Affiliations:** ^1^ Department of Neonatal Intensive Care Unit, The First Affiliated Hospital of Zhengzhou University, Henan, China; ^2^ Department of Cardiothoracic Vascular Surgery, The First Affiliated Hospital of Zhengzhou University, Henan, China; ^3^ Extracorporeal Support Center, The First Affiliated Hospital of Zhengzhou University, Henan, China; ^4^ Department of Cardiology, The First Affiliated Hospital of Zhengzhou University, Henan, China; ^5^ Department of Pediatric Intensive Care Unit, The First Affiliated Hospital of Zhengzhou University Henan, Henan, China

**Keywords:** congenital tracheal stenosis (CTS), tracheal reconstructive surgery, extracorporeal membrane oxygenation (ECMO), premature infants, very low birth weight, high-risk treatment, bronchoscopy

## Abstract

**Background:**

Congenital tracheal stenosis (CTS) is a rare but life-threatening malformation of the trachea. Surgical reconstruction is the treatment of choice in symptomatic cases which is highly risky and is rarely performed in extremely premature infants. With this, reporting a case of CTS managed by tracheal reconstructive surgery under ECMO in a baby weighing 1.47 kg at 32 + 1 WOG was the first ever case in China.

**Case presentation:**

A premature newborn with a very low birth weight (VLBW) was admitted to our institute for breathing difficulties, requiring mechanical ventilation, and experienced two unsuccessful attempts of extubation. The team performed tracheal reconstructive surgery supported by ECMO after identifying lower tracheal stenosis through a bronchoscopy examination. One month after the surgery, oxygen support was able to discontinue. The patient's entire hospitalization was incredibly challenging, marked by hemodynamic instability with persistent anemia, and disseminated intravascular coagulation (DIC), which were managed with great care. Despite the difficult stay, a follow-up bronchoscopy revealed no obstruction or tracheal stenosis, leading to a successful discharge.

**Conclusion:**

Advancements in diagnostic techniques and innovative management methods have made diagnosing and treating CTS easier, even in premature infants. Our case is the first in China to successfully undergo tracheal reconstructive surgery supported by ECMO, inspiring future achievements in the medical field.

## Introduction

Congenital tracheal stenosis (CTS) is a rare disease, with an estimated incidence rate of 1/64,500 (1) and high mortality rate. Bronchoscopy can help to diagnose neonates with congenital airway developmental problems because their symptoms are nonspecific (2, 3). This article reports a successful case of CTS managed by tracheal reconstructive surgery in a premature infant in China, demonstrating the importance of multidisciplinary collaboration in treating such a rare condition.

## Case presentation

We present a case of a premature male infant on the 8th Day of Life (DOL), referred to our institute with chief complaints of difficulty in breathing soon after birth. The baby was delivered via emergency cesarean section by 3rd gravida and 2nd para mother due to severe eclampsia, placenta previa in fetal distress at 30 + 1 weeks of gestation (WOG) weighing 1,300 g, and with an umbilical cord wrapped around the neck twice. The APGAR scores were 2 and 3 at 1 and 5 min, respectively. Consequently, the infant received neonatal resuscitation with bag and mask ventilation, and was subsequently intubated, and was transferred to the NICU. Additionally, the baby was administered two doses of pulmonary surfactants (PS) in conjunction with broad-spectrum antibiotics for 7 days. Despite treatment and ventilator assistance, the baby's respiratory distress worsened, prompting the parents to seek further management elsewhere. Upon arrival to our department, the baby was lethargic, pale, slightly jaundiced, and exhibiting tachypnea. A systemic examination revealed respiratory distress, a normal heart rate, and slight hepatomegaly, indicating signs of cardiac failure. The parents disclosed the absence of any reported specific or genetic diseases and no consanguineous marriage.

## Diagnosis

**Figure 1:**
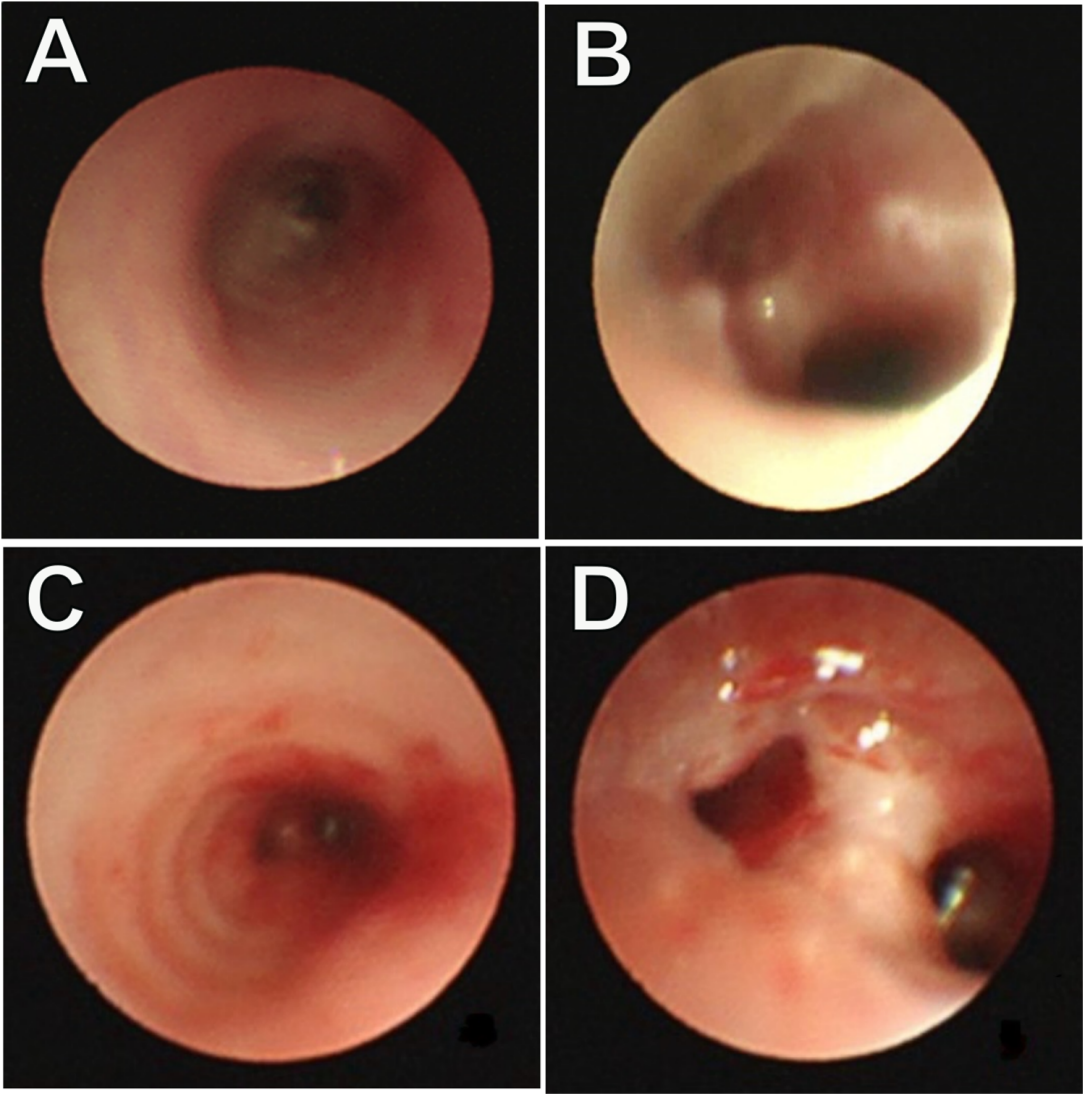
Bronchoscopy examination: **(A)** stenosis of the lower segment of the trachea; **(B)** the narrow lumen has an irregular margin, and no membrane protrusion. Repeated bronchoscopy examination. **(C)** An unobstructed lower segment of the trachea; **(D)** Anastomotic opening with no granulation tissue area, and no signs of tracheal narrowing.

**Figure 2:**
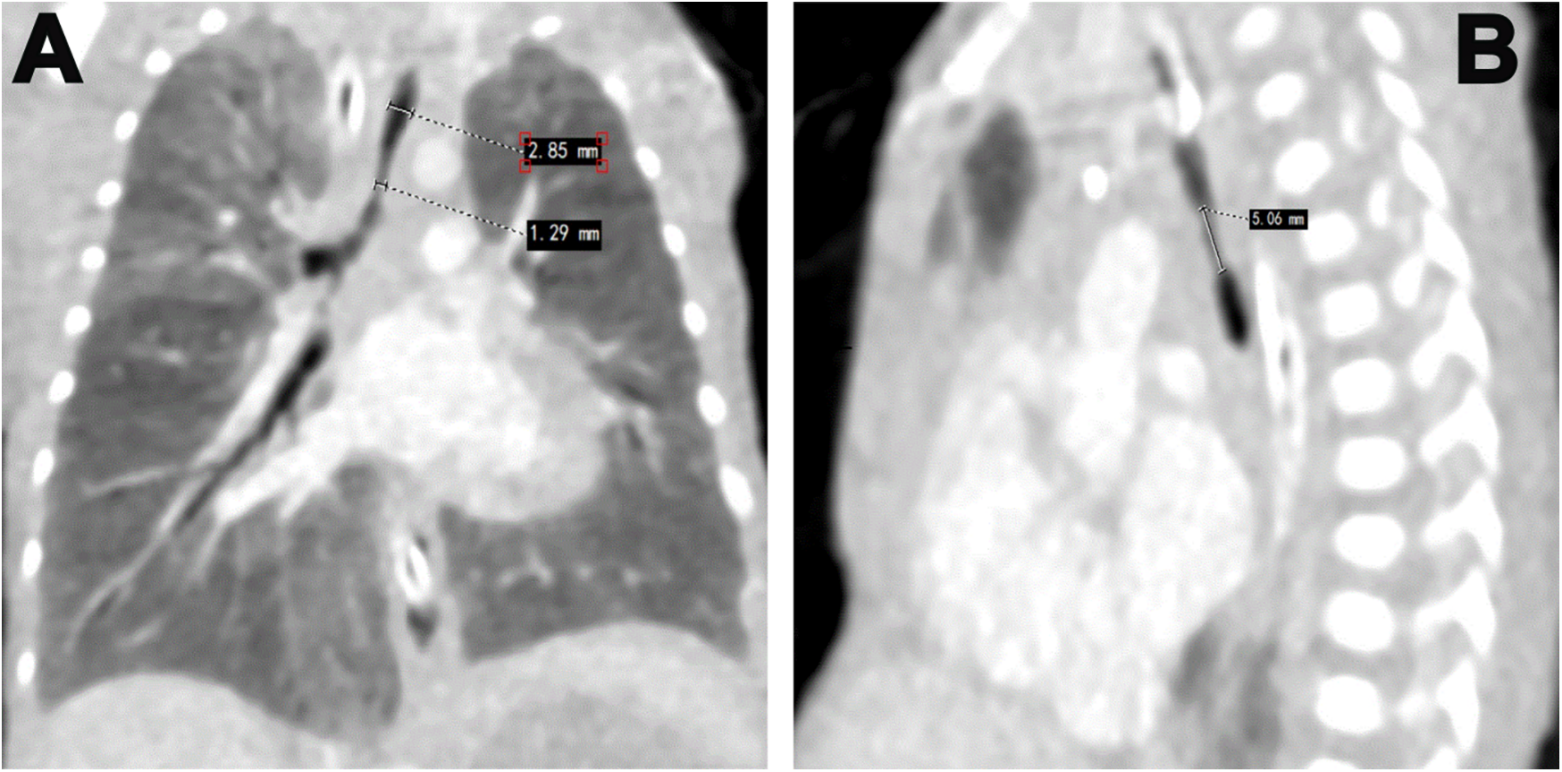
Enhance CT images showing: **(A)** the upper width of the trachea is 2.9 mm whereas the stenosed part has a width of 1.9 mm. **(B)** showing a length of stenosed part 5.06 mm.

Our patient underwent various sets of investigations to diagnose this rare condition and prepare for the challenges of high-risk treatment. The initial chest x-ray revealed increased lung markings and bilateral lung inflammations. Arterial blood gas (ABG) analysis indicated severe mixed metabolic and respiratory acidosis, pointing to respiratory failure, which necessitated adjustments to the mechanical ventilator (MV) parameters. Additionally, PS was readministered at our hospital. Despite these adjustments and ABG monitoring, two attempts at extubation were unsuccessful. Subsequently, the team performed a flexible bronchoscopy which revealed severe tracheal stenosis (Figures 1A,B), correlated by enhanced CT chest (Figure 2). The upper width of the trachea was 2.9 mm while the stenosed part had a width of 1.9 mm and a length of 5.06 mm. CT angiography showed no vascular malformations or thoracic tumors. A multidisciplinary consultation was recommended for tracheal reconstructive surgery under general anesthesia (GA).

The preliminary tests indicated acute infections, as evidenced by elevated white blood cell (WBC) and neutrophil counts, and decrease hemoglobin and platelet counts. Cerebrospinal fluid (CSF) analysis confirmed intracranial infections, showing elevated nucleated cells, total CSF protein, and albumin levels. Consequently, intravenous antibiotics and antifungals were administered to address the infections. Additionally, the patient exhibited high NT-proBNP levels, and significant hepatomegaly suggesting cardiac failure, accompanied by a patent ductus arteriosus, which was managed conservatively.

## Surgical intervention

At corrected gestational age of 32 weeks and 1 day, the patient, weighing 1,470 g, underwent tracheal reconstructive surgery under GA. The child was positioned on the left side, and an 8 cm incision was made at the right fourth intercostal space to access the thoracic cavity. This allowed the establishment of a three-dimensional extracorporeal membrane oxygenation (ECMO) pathway, with an 8Fr arterial cannula inserted into the ascending aorta and a 14Fr venous cannula placed in the right atrium. The machine was operated at a rotationalspeed of 1,700 rpm, a flow rate of about 0.25l/min, ventilation volume of about 1l/min, and a maintenance duration of approximately 200s, with oxygen saturation of 98%. A segmental resection of the narrowed trachea (Length-5.06 mm) was excised followed by an end-to-end anastomosis with continuous suture by a non-absorbable threads. In this premature infant, the stenosed part of the trachea was relatively modest reducing the risk of a figure of 8 deformities. Approximately 2.5 h after the surgery concluded, ECMO support was discontinued and the patient was transferred to the NICU for additional care.

## Postoperative management

It is a critical aspect of patient care to ensure the head and neck are positioned at a 15–30° forward angle with the axis aligned to reduce tension at the anastomotic site. Meanwhile, respiratory support is of paramount importance. On the fourth postoperative day (POD), blood clots were frequently observed in the endotracheal (ET) tube due to impaired coagulation functions leading to tube obstruction. Hence, such clots were removed under the guidance of direct laryngoscope visualization, employing negative pressure suction through a 6.0Fr suction tube at 80 mmHg for less than 6 s. Despite the inability to ascertain the exact bleeding source, the endotracheal tube was removed, and the patient was then placed on synchronized nasal intermittent positive pressure ventilation (SNIPPV), ultimately moving to a humidified high-flow nasal cannula on 7th POD. This approach led to no further bleeding and improved the patient's oxygen saturation, allowing for the cessation of oxygen therapy a month post-surgery. Additionally, in accordance with the DART protocol (4), a brief low-dose dexamethasone course was administered to lessen the broncho-pulmonary dysplasia (BPD), a major complication during the hospitalization.

**Figure 3:**
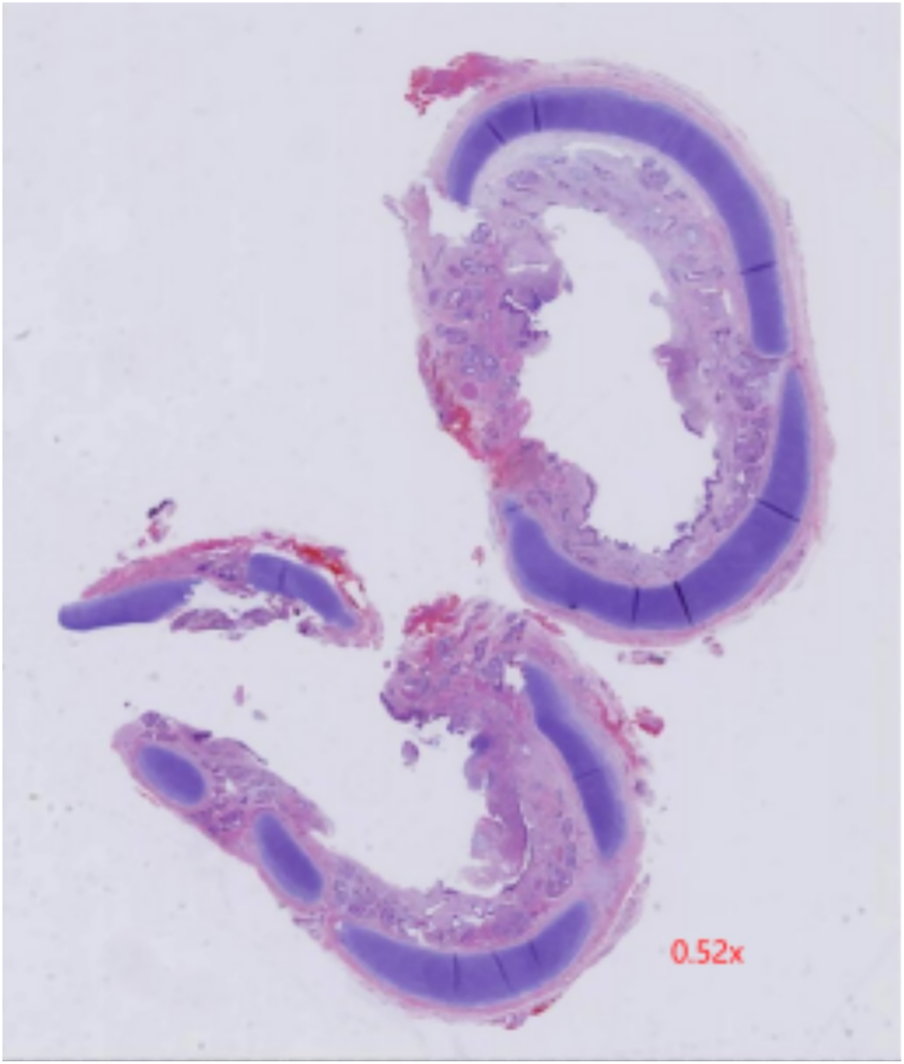
Postoperative pathology showed chronic inflammation of bronchial mucosa, polypoid hyperplasia, and small calcification in the tracheal mass along with its margin.

Furthermore, eight hours post-surgery, the patient developed paroxysmal supraventricular tachycardia (PSVT) with a peak heart rate of 225 beats per minute. An ice pack vagal maneuver was attempted twice, however was ineffective. Adenosine was subsequently administered at dosages of 100 μg/kg, 200 μg/kg, and 400 μg/kg at 2-minute intervals, resulting in a reduction in the pulse rate to 190–180 beats per minute. However, PSVT reappeared shortly after and was treated with a continuous infusion of amiodarone, resulting in the restoration of sinus rhythm within 24 h. Subsequent monitoring of coagulation functions revealed prolonged PT and APTT, alongside reduced fibrinogen and platelet levels. These issues were addressed by administering FFP, cryoprecipitate, and packed RBCs multiple times. A fluid restriction of 150 ml/kg/day was enforced to control volume overload, and rigorous infection prevention techniques were implemented. Early parenteral feeding and nasogastric tube feeding were initiated for nutritional support. Cranial ultrasonography was performed every third day due to the patient's grade II intracranial bleeding prior to surgery and requiring postoperative monitoring. The brain MRI identified cerebral hemorrhage without significant changes, hence, the situation was controlled with consistent weekly administrations of vitamin K. One month following the surgery, the child had significantly recovered. A follow-up bronchoscopy revealed a clear tracheobronchial tree with no granulation tissue or signs of tracheal stenosis (Figures 1C,D). In addition, postoperative pathology showed chronic inflammation of bronchial mucosa, polypoid hyperplasia, and small calcification in the tracheal mass along with its margin (Figure 3). Consequently, the child was discharged weighing 2.53 kg, stable in room air, self-sucking, under demand feeding.

The child was readmitted a month after discharge due to pale lips and complexion persisting for 3 days and was diagnosed with iron deficient anemia. He received a packed RBC transfusion and iron supplements. A brain MRI showed subacute hemorrhage in the body and posterior horn of the left ventricle. Neurologists recommended close monitoring and a 7-day course of Vitamin K injections to address the bleeding. Hence, the patient was discharged following conservative management. Currently, the child is 2 years and 9 months old, and has undergone regular follow-ups every six months, which showed no issues with hearing or fundus examinations, no significant alterations in the brain MRI, normal growth and development, and a moderate Peabody developmental motor score.

## Discussion

Wolman's 1941 (5) was the first to describe CTS. As the disease was not yet widely known, blind conservative treatment was adopted initially which led to a long-term dependency on ventilators, potentially inducing ventilator-associated pneumonia, respiratory failure, and even high mortality rates (6). With the increasing prevalence of bronchoscopy and CT scans, it has gradually gained recognition (7). Upper airway malformation narrows or obstructions the respiratory tract above the cricoid cartilage for numerous reasons, causing dyspnoea, cyanosis, stridor, and problem feeding immediately after birth (8). Our case, born at 30 + 1 WOG, presented with similar syndromes after birth. After unsuccessful extubation attempts, a bronchoscopy examination ruled out the possibility of fatal congenital airway malformation.

Three surgical techniques are often employed to address CTS: (1) end-to-end anastomosis with immediate excision of the narrowed section, (2) slide tracheoplasty, and (3) patch tracheoplasty. Surgery is the standard treatment for CTS patients; however, asymptomatic children often receive conservative management. Long-segment stenosis often necessitates slide tracheoplasty, while short-segment stenosis may be addressed with resection or anastomosis. There is no global consensus on the optimal treatment approach for CTS in newborns. Individualization should lead to CTS therapy and surgical intervention should be scheduled based on the specific circumstances of each center. CTS was firmly diagnosed utilizing a bronchoscopy examination and an enhanced CT scan to rule out the presence of external pressure, vascular rings, or tumors. The interdisciplinary panel recommended ECMO during surgery under GA for reconstructive surgery citing potential risks such as airway damage, ventilator-associated infections, BPD, and pulmonary arterial hypertension in newborns undergoing tracheal repair, which may have a mortality rate of 70% (9). The success of this case has set the record for the first time in China enabling us to fill gaps in the implementation of ECMO for preterm newborns with VLBW.

Limited national and international studies exist on the treatment of neonatal CTS, and the most effective strategy is the best perioperative care and surgical technique remain undermined. Hartnick et al. (10) reported that premature infants with a gestational age of 26 weeks weighing 750 g underwent MV maintenance treatment after balloon dilation until their body weight reached 3 kg, at which point resection and anastomosis were performed. A case of premature infants, aged 25 + 5 weeks, weighing 860 g at birth and 950 g one month later, underwent slide tracheoplasty without the need for ECMO support, as documented by Zibdawi et al (11). Furthermore, Yong et al. (12) described a premature infant born at 29 weeks, weighing 1,049 g, tracheoplasty was performed with ECMO support after achieving on 2.5 kg body weight of the baby at 2 months of age. ECMO in newborns is mainly employed for severe respiratory failure, sepsis, congenital diaphragmatic hernia, cardiac surgery, tracheal stenosis, etc. (13), to sustain gaseous exchange throughout the reconstructive surgery to provide a clearer anatomical field of view for delicate surgery. Premature infants with small airway diameters encounter the most obstacles before CTS surgical intervention: awaiting airway expansion, optimizing nutritional status, and controlling infections (14).

Economic and prognostic factors delay the advancement of ECMO technology in China, while cannulation for ECMO was challenging due to small blood vessels and preterm infants’ low body weight. Ultrasound is used for the assessment of VLBW index. We performed the central catheterization (right atrium aorta), ascending aorta 8Fr arterial canulation, and right atrial 14Fr venous cannulation which promotes a decrease in the incidence of cerebral hemorrhage and mortality for premature newborns receiving ECMO therapy (15).

The Guidelines for neonatal respiratory failure outline the subsequent relative contraindications for ECMO assistance in newborn respiratory failure: (1) irreversible organ damage (unless organ transplantation is being considered); (2) body weight less than 2 kg; gestational age less than 34 weeks; (3) mechanical ventilation for more than 14 days (16);. Fallon et al. (17) reported that ECMO was used to ensure the survival of all premature infants weighing under 2 kg (1.5 kg, 1.6 kg, and 1.8 kg), with certain facilities have achieved a minimum body weight of 1 kg (18) lowering the gestational age limit. The use of ECMO may be extended to more premature infants in the upcoming years (13). Our case illustrates that at 32 + 1 weeks, weighed 1.47 kg only and was under MV support for more than 14 days prior to surgery. Consequently, we must state that our case has overcome all those challenges by successfully discharging the child and maintaining follow-ups. Advancements in contemporary diagnostic procedures and innovative management modalities are continually enhancing the prognosis of these premature infants (19). Newborns should undergo a thorough preoperative evaluation, focusing on high-risk aspects like bleeding. Rapid response to massive bleeding requires volume management and lung protection breathing. Postoperative management of infections, bleeding disorders, and respiratory needs interdisciplinary teamwork.

## Conclusion

Recent medical advancements and innovative techniques have enhanced CTS more diagnosable and treatable, particularly in neonates with low birth weight. Multidisciplinary consultations are crucial for optimal treatment, and our case serves as an example for future healthcare professionals.

## Data Availability

The raw data supporting the conclusions of this article will be made available by the authors, without undue reservation.
